# Generation of Micronuclei during Interphase by Coupling between Cytoplasmic Membrane Blebbing and Nuclear Budding

**DOI:** 10.1371/journal.pone.0027233

**Published:** 2011-11-02

**Authors:** Koh-ichi Utani, Atsushi Okamoto, Noriaki Shimizu

**Affiliations:** Graduate School of Biosphere Science, Hiroshima University, Higashi-hiroshima, Hiroshima, Japan; Brunel University, United Kingdom

## Abstract

Micronucleation, mediated by interphase nuclear budding, has been repeatedly suggested, but the process is still enigmatic. In the present study, we confirmed the previous observation that there are lamin B1-negative micronuclei in addition to the positive ones. A large cytoplasmic bleb was found to frequently entrap lamin B1-negative micronuclei, which were connected to the nucleus by a thin chromatin stalk. At the bottom of the stalk, the nuclear lamin B1 structure appeared broken. Chromatin extrusion through lamina breaks has been referred to as herniation or a blister of the nucleus, and has been observed after the expression of viral proteins. A cell line in which extrachromosomal double minutes and lamin B1 protein were simultaneously visualized in different colors in live cells was established. By using these cells, time-lapse microscopy revealed that cytoplasmic membrane blebbing occurred simultaneously with the extrusion of nuclear content, which generated lamin B1-negative micronuclei during interphase. Furthermore, activation of cytoplasmic membrane blebbing by the addition of fresh serum or camptothecin induced nuclear budding within 1 to 10 minutes, which suggested that blebbing might be the cause of the budding. After the induction of blebbing, the frequency of lamin-negative micronuclei increased. The budding was most frequent during S phase and more efficiently entrapped small extrachromosomal chromatin than the large chromosome arm. Based on these results, we suggest a novel mechanism in which cytoplasmic membrane dynamics pulls the chromatin out of the nucleus through the lamina break. Evidence for such a mechanism was obtained in certain cancer cell lines including human COLO 320 and HeLa. The mechanism could significantly perturb the genome and influence cancer cell phenotypes.

## Introduction

Growing mammalian cells often form secondary nuclei that are smaller than the main nucleus and that are referred to as micronuclei. Usually, micronuclei are generated from acentric chromosomal fragments or malsegregated whole chromosomes after mitosis. Such chromatin is left behind the separating chromosomes during anaphase, and generates micronuclei independently from the main nucleus at the following interphase. Acentric chromosomal fragments may be derived from unrepaired or miss-repaired chromatin after DNA double strand breakage, while malsegregated whole chromosomes can arise from chromosomes that are not bound to the spindle. The latter can occur by several mechanisms including changes in the DNA methylation level at the centromeric region (reviewed in ref. [1]). The malsegregation of chromosomes may also occur when they are merotelically bound to microtubules coming from both spindle poles [2]. In addition, the micronucleus may be formed from the chromatin bridge between segregating sister chromatids, if the bridge breaks at multiple sites during the anaphase to cytokinesis transition [3]-[5]. Chromatin bridge formation can be caused by the miss-repair of DNA damage, and is involved in the breakage-fusion-bridge (BFB) cycle that destabilizes the chromosome arm and amplifies the genes critical to cancer cell growth [6], [7]. The appearance of micronuclei is closely linked to the DNA damage-repair process and genome instability, and monitoring the frequency of micronuclei is therefore widely used to assess the environmental or endogenous stresses that damage the genome and cause cancer (for a review, see the special issue of *Mutagenesis*, vol 26, no. 1, 2011).

Micronuclei are generated not only from chromosomal materials, but also from extrachromosomal elements. Extrachromosomal elements called double minutes (DMs) are cytogenetic manifestations of gene amplification that are detected in many human cancer cells. The amplified genes on DMs determine the malignant phenotype of cancer cells; therefore the elimination of DMs from cancer cells results in the loss of malignant phenotypes [8]–[10]. The elimination of DMs is mediated by their specific incorporation into micronuclei, and the purification of such micronuclei yielded highly purified DM DNA [11]. The mechanism of the generation of such DM-type micronuclei is related to the intracellular behavior of DMs during cell cycle progression (reviewed in ref. [12], [13]). Namely, acentric DMs are segregated to daughter cells by sticking to the centric chromosome arm during mitosis [14]–[16]. These DMs localize to the nuclear periphery during the G1 phase, and move to the interior during the early S phase, when DMs themselves are replicated [17], [18]. During the early S phase, the presence of low concentrations of hydroxyurea (HU) induces DNA damage at the replication site. The damage at the chromosome arm is repaired rapidly, but difficulties encountered in the repair of DNA damage at DMs induce their aggregation [19]. The aggregated DMs lag behind the separating chromosomes at anaphase and generate DM-type micronuclei. This mechanism may be applied to a broad spectrum of extrachromosomal elements, because many kinds of viral nuclear plasmids stick to the chromosome arm during mitosis (reviewed in ref. [12], [15]).

Micronuclei resemble nuclei in structure, and a large portion of them has nuclear lamina. However, lamin-negative micronuclei were reported for both chromosome-type [3], [20]–[22] and DM-type micronuclei [16], [22]. The presence or absence of lamin around micronuclei has important implications for the phenotypes of cells, because it is correlated with transcription [22] or replication (Okamoto et al., unpublished data) inside micronuclei. Investigation of the origin of micronucleus-heterogeneity is therefore an important task. This heterogeneity could be attributed to differences in the origin of micronuclei, as chromosome-type lamin B-positive micronuclei are generated from the anaphase laggards, while lamin B-negative micronuclei are generated from the anaphase chromatin bridge [3]. The present report describes another mechanism involved in the generation of lamin-negative micronuclei, namely interphase nuclear budding.

In addition to the mitotic generation of micronuclei, the formation of micronuclei during interphase through nuclear budding has been repeatedly hypothesized based on the detection of nuclear buds or protrusions in cytogenetic preparations (reviewed in ref. [23]), and the close resemblance of some of them to micronuclei, with the exception of their connection to the nucleus through a chromatin stalk. Furthermore, a cell-cycle synchronization experiment suggested that a portion of DM-type micronuclei might be generated through nuclear budding [24]. The generation of micronuclei through a budding process in the mammalian nucleus, which is reinforced by the nuclear lamina, is surprising. Prior work based on the simultaneous visualization of DMs and the lamin protein did not support the protrusion of a portion of the nucleus with lamina as a mechanism for the generation of buds/micronuclei [16]. Instead, lamin-negative DM aggregates were detected on the outside of the nuclear lamina in a shape resembling a bud. The present study shows that the budding structure was generated through a break in the lamina, which resembled the nuclear blister induced by the HIV Vpr protein [25] or the herniation induced by the reoviral sigma 1s protein [26]. Extrachromosomal small DMs frequently localize to the nuclear periphery [17], and can pass more easily through the break in the lamina than the large chromosomal arm. Furthermore, time-lapse live cell imaging of DMs, lamin B1 and DNA revealed that budding is coupled to cytoplasmic membrane blebbing. Blebbing is frequently associated with cell locomotion, and is activated during mitosis or apoptotic cell death. The present study is the first to link two previously unrelated phenomena, namely the elimination of nuclear materials and cytoplasmic membrane blebbing.

## Results

### Lamin B1-negative micronuclei coexist with postmitotically generated lamin B1-positive micronuclei

**Figure 1:**
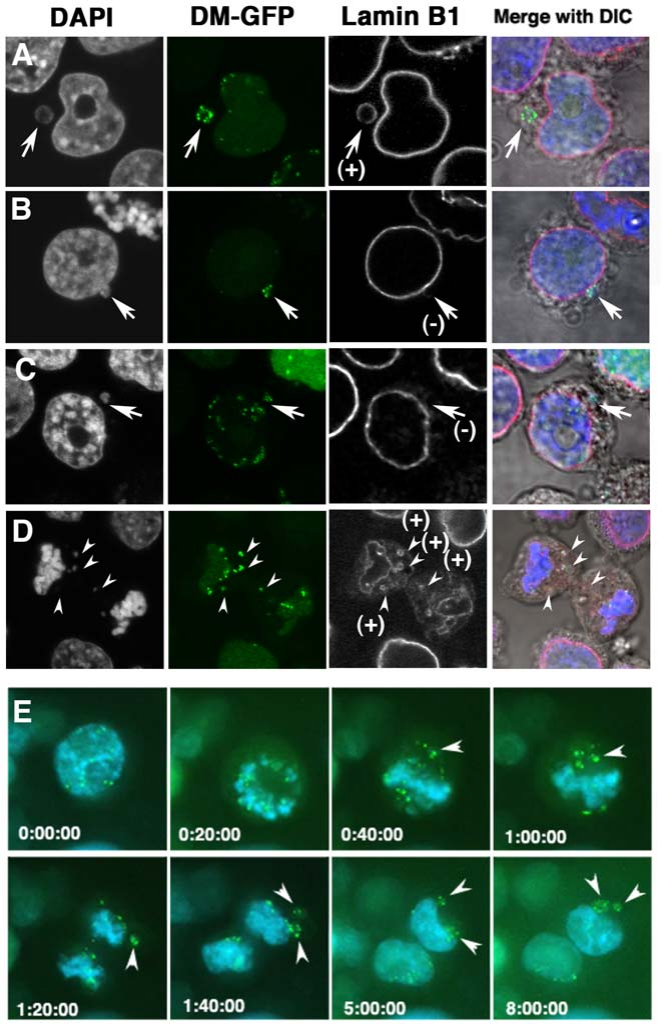
Lamin B1-negative micronuclei and postmitotically-generated lamin B1 positive micronuclei were detected. (A to D) COLO 320DM-GFP cells were fixed with PFA and the lamin B1 protein was detected by immunofluorescence. Representative confocal images of the DM-type micronucleus with lamin B1 (A; arrow) or without lamin B1 (B and C; arrow). The image of lamin B1 is shown in gray-scale, and it was shown in red in merged panels. The micronucleus in B is attached to the nucleus whereas the one in C is detached from the nucleus. In mitotic cells in telophase, the aggregated DMs were left behind the separating chromatids, and lamin B1 was detected at the rim (D; arrowheads). (E) Living COLO 320DM-GFP cells were stained with Hoechst 33342 and analyzed by time-lapse microscopy (E). DM aggregates were located separate from chromosomes at metaphase and anaphase, and generated the DM-type micronuclei after mitosis (arrowheads). Elapsed time (in hours:minutes:seconds) is shown in each images.

The human COLO 320DM-GFP cell line, in which LacO-tagged DMs were visualized by the expression of the LacR-GFP fusion protein, was previously established (see the methods section). This cell line enables the detection of DMs without the use of FISH, which requires heat denaturation and may disrupt the 3-D structure of the cells. Fixation of these cells by PFA followed by immunofluorescence-based detection of lamin B1 protein revealed the presence of DM-enriched micronuclei surrounded by lamina (Figure 1A) and those without lamina (Figure 1B, C), as previously reported [16], [22]. The use of a cell line bearing visible DMs enabled the application of time-lapse imaging to examine the formation of DM-enriched micronuclei. As shown in Figure 1E, the aggregated DMs detached from the chromosomes during the metaphase to anaphase transition, and formed micronuclei after the end of mitosis. The time-lapse observation confirmed the hypothesis derived from fixed cell observation that micronuclei arise from aggregated DMs after mitosis [16], [19]. Furthermore, simultaneous visualization of lamin B1 and DMs in fixed cells showed that micronuclei generated through this mechanism are surrounded by Lamin B1 (Figure 1D).

### Large cytoplasmic blebs are associated with nuclear budding through breaks in the lamina

**Figure 2:**
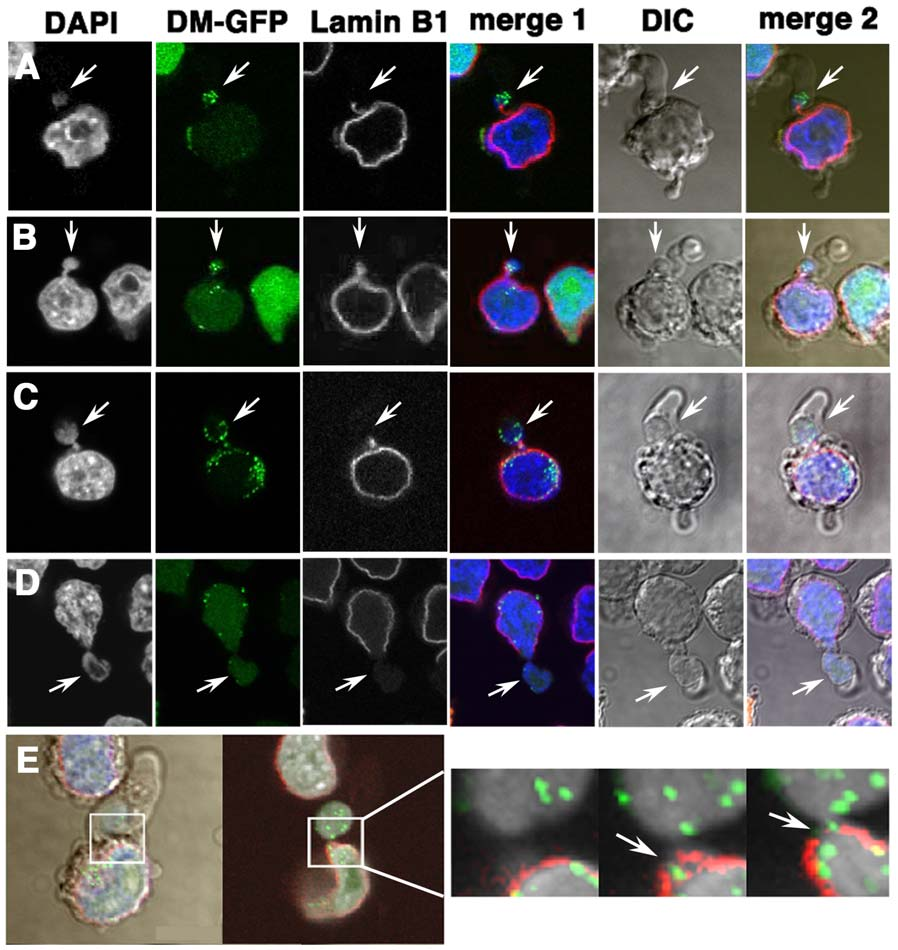
The large cytoplasmic bleb entraps the nuclear bud/micronucleus that is devoid of lamin B1. (A to D) COLO 320DM-GFP cells were fixed, and the lamin B1 protein was detected as in Figure 1. Representative confocal images of lamin-negative micronuclei (white arrows) connected to the nucleus by thin chromatin are shown. These micronuclei were inside the large cytoplasmic bleb. (E) The left two panels show the merged images of lamin B1 (red), DMs (green), DAPI (blue in left, gray in right) and DIC (gray in left). The rectangle region was enlarged in the right three panels showing the serial confocal images taken at 0.8 µm intervals in the Z-axis. The break in the lamin B1 envelope is indicated by a white arrow.

Cultured COLO 320DM cells and COLO 320DM-GFP cells did not spread on the substratum, but rather attached weakly to the tissue culture coated dish. The surface of these round-shaped cells had many protrusions, which are commonly referred to as cytoplasmic membrane blebbing. Imaging of the blebbing of rounded cells in addition to labeled DMs, lamin B1 and DAPI-stained DNA by DIC microscopy revealed that, surprisingly, the large cytoplasmic membrane protrusions entrapped the lamin B1-negative micronuclei. For simplicity, the large cytoplasmic membrane protrusion is hereafter referred to as a “large (cytoplasmic) bleb”. The micronuclei in the large bleb contained highly concentrated DMs (Figure 2A and B) or DMs with other chromosomal material (Figure 2C). Notably, a DAPI-stained thin chromatin stalk connected a portion of this micronucleus to the nucleus (Figure 2B and C). These types of micronuclei are referred to as “the nuclear buds” during the micronucleus test (for a review, see, ref. [27]). The lamin B1 protein was not detected at these micronuclei, but it showed dense staining at the stalk between the micronucleus and the nucleus, regardless of whether or not the chromatin was microscopically visible at the stalk (Figure 2A to C). There were large buds showing the protrusion of large amounts of chromatin from the nucleus (Figure 2D), and these buds were associated with obvious breaks in the nuclear lamina. The morphology of the nuclear bud was strikingly similar to that of the phenomenon reported as nuclear herniation [26] and to the nuclear blister [25], which are induced by viral proteins. Furthermore, careful observation of the cells by confocal microscopy showed that the lamin-negative micronuclei inside the large cytoplasmic blebs were frequently associated with small lamina breaks, as shown in Figure 2E. These results indicate that the large cytoplasmic bleb is correlated with the extrusion of nuclear content through lamina breaks, which may explain the so-called “nuclear budding” phenomenon previously described [24], [27].

### Fresh serum efficiently induced both the large cytoplasmic blebs and the nuclear budding/micronucleation

**Figure 3:**
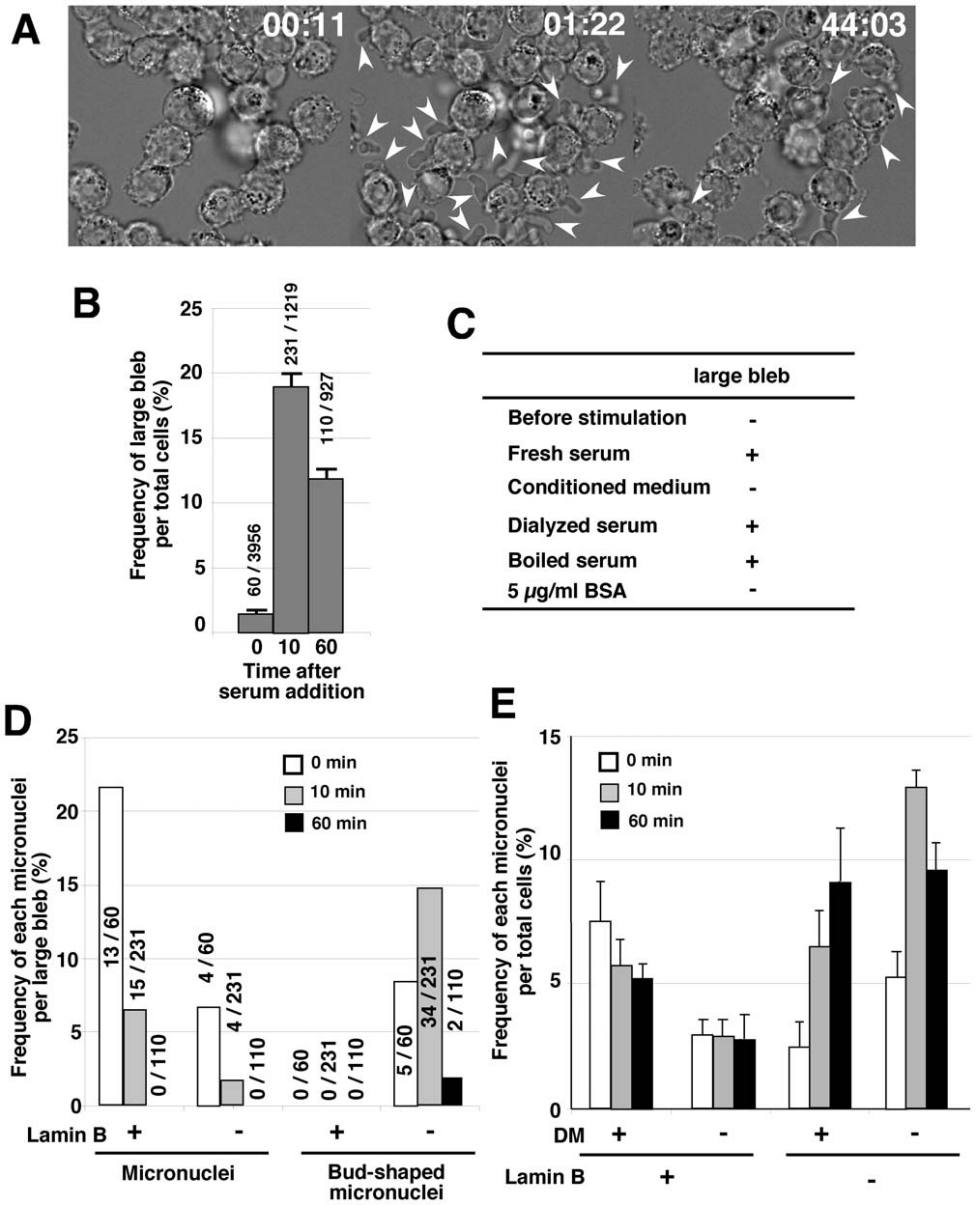
Fresh serum induced the formation of large blebs and lamin B1-negative micronuclei. (A) DIC images of living COLO 320DM-GFP cells are shown. The incubation time (min:sec) after the addition of fresh serum is shown in each panel. The arrows indicate the large blebs. (B) The cells were fixed with PFA and the frequency of the large blebs among the total cells was measured. (C) Cells cultured for two days in a medium containing 10% serum rarely showed the large bleb (-; “before stimulation”), whereas addition of fresh serum or treated serum at the final concentration of 10% induced extensive blebbing (+) after 10 min. (D) Cells were stimulated with fresh serum for the indicated time and fixed for detection of the lamin B protein. The frequency of the formation of the large bleb containing the micronuclei that were apart from the nuclei or the bud-shaped micronuclei that was connected to the nuclei among the total number of large blebs was calculated in 300 to 1,000 cells. The incidences among the counted blebs were noted in the graph. (E) In the same slides used in D, the frequencies of the micronuclei with/without DMs or with/without lamin B1 among the total cells were measured by examining 1,000 cells in three replicates. Error bars represent mean +/- SEM.

The frequency of the large cytoplasmic blebs was low among the logarithmically growing cells (see below). The addition of fresh serum has been reported to induce cytoplasmic membrane blebbing [28], [29]. In the present study, the addition of fresh fetal calf serum (FCS) to the logarithmically growing cells efficiently induced the formation of large cytoplasmic blebs in the COLO 320DM-GFP cells within a few minutes (Figure 3A and B), which was then followed by a decrease in their frequency up to 60 min. (Figure 3B). The bleb-inducing activity was detected in the fresh serum, but not in the serum-containing medium used for cell growth (the “conditioned medium” in Figure 3C). The activity was associated with the heat-resistant non-dialyzable fraction, and it was not associated with bovine serum albumin (BSA; Figure 3C).

Because serum induced the large cytoplasmic blebs, the frequency of micronuclei per large bleb decreased after the serum addition (Figure 3D). On the other hand, measurement of the frequency of the large cytoplasmic blebs that entrapped “the bud-shaped micronuclei”, namely those connected to the nucleus by a thin chromatin stalk, during the course of serum activation (Figure 3D) showed that the frequency increased until 10 min after serum addition and declined until 60 min (Figure 3D). As shown in the graph, these micronuclei with stalks were always lamin B1-negative, which is shown in the representative image in Figure 2. The frequency of the total intracellular micronuclei was measured independently of their connection to the nucleus by the stalk or their entrapment in cytoplasmic blebs (Figure 3E). The results showed that the frequency of lamin B1-negative micronuclei increased after fresh serum addition, whereas the frequency of lamin B1-positive micronuclei did not show a change. This increase was observed for both DM-type and chromosome-type (DM-negative) micronuclei. Taken together, these results indicate that nuclear budding is associated with the formation of micronuclei during interphase.

Although cytoplasmic membrane blebbing is known to become active during the initial phase of apoptosis, the present results show that fresh serum induced blebbing but did not cause the appearance of apoptotic cells (Figure S1). Therefore, the cytoplasmic blebbing and nuclear budding induced by fresh serum were not related to apoptosis.

### Time-lapse observation revealed that the process of interphase micronucleation through nuclear budding was coupled with cytoplasmic blebbing

**Figure 4:**
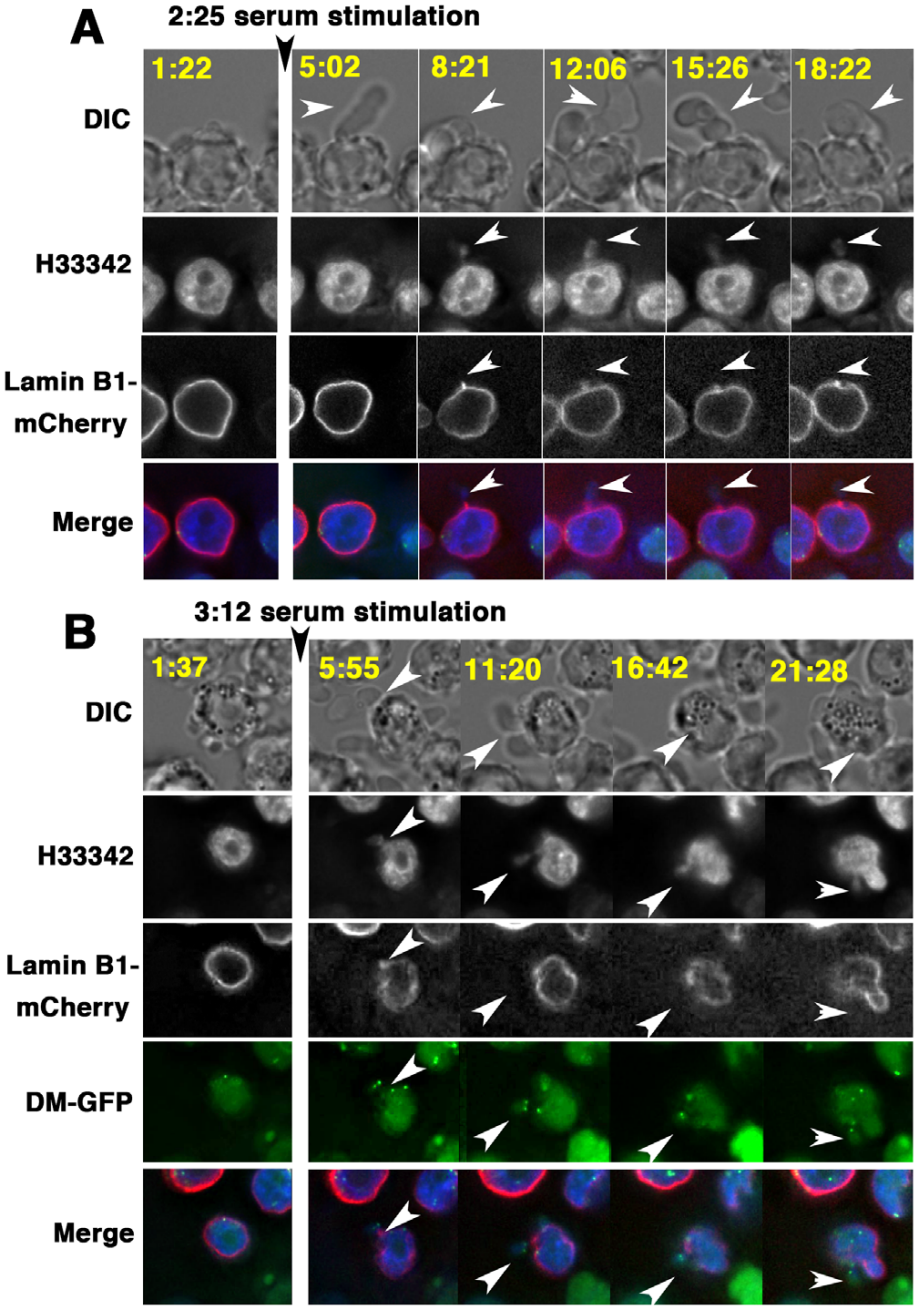
Time-lapse microscopy indicated that nuclear budding is coupled to cytoplasmic blebbing, which generates lamin-negative micronuclei. (A and B) The DNA in living COLO 320DM-GFP/lamin B1-mCherry cells was stained with Hoechst 33342 for time-lapse observation before and after fresh serum stimulation. The images were obtained at 3 min (A) or 5 min (B) intervals. The images corresponding to DM-GFP were omitted in A because the nuclear budding did not contain the DM-GFP signal. Elapsed time (in minutes:seconds) after the start of the experiment is shown in each image. White arrowheads indicate the cytoplasmic blebbing and nuclear budding.

The appearance of the large cytoplasmic bleb that entrapped the lamin-negative micronuclei indicated that two possible mechanisms may exist: the blebbing and the formation of micronuclei could be coupled, or the bleb may entrap previously-generated micronuclei. To examine these possible mechanisms, time-lapse microscopic observation of COLO 320DM-GFP cells (Figure S2, S3, Movie S1) or COLO 320DM-GFP/lamin B1-mCherry cells (Figure 4 and Figure S4) was performed. The cells appearing in these representative images had no micronucleus before the addition of fresh serum. The addition of fresh serum induced the appearance of the large cytoplasmic bleb within a few minutes, which was oriented in the x-y plane (Figure 4A) or slanted to the z-axis (Figure 4B). Importantly, the nuclear buds appeared inside the cytoplasmic bleb at the same time with the blebbing (Figure 4B) or just after the blebbing (Figure 4A). These buds contained DMs (Figure 4B) or not (Figure 4A; the images for DM-GFP are not shown). Images stained for the detection of DNA (H33342) showed the nuclear buds connected to the nucleus by the chromatin stalk. The images with lamin B1-mCherry staining showed that the protein was not located at the nuclear buds, but located heavily at the chromatin stalk. These structures were identical to the structures observed in the fixed cells (Figure 2). These time-lapse observations clearly showed that the formation of the nuclear bud is concurrent with the generation of the large bleb by the cytoplasmic membrane at the position of the nuclear buds, which indicates that these events are interrelated. Hereafter, this phenomenon is referred to as “the (cytoplasmic) blebbing and the (nuclear) budding”.

### Induction of blebbing and budding by fresh serum is dependent on the cell lines and the cell-to-substratum adherence

**Figure 5:**
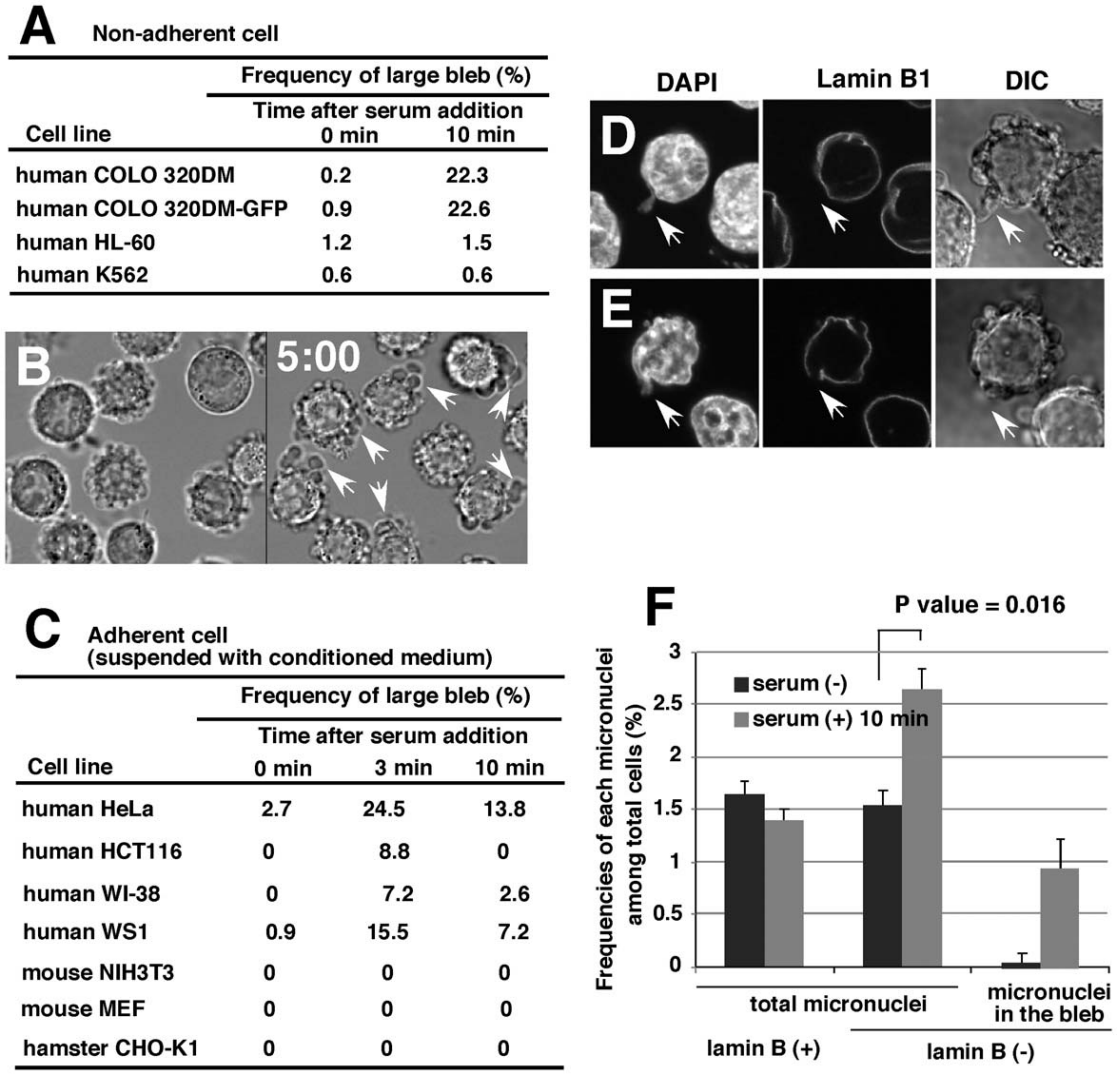
Induction of blebbing and budding by fresh serum was dependent on the cell line and the cell-to-substratum adherence. (A) Several non-adherent cell lines were stimulated by fresh serum, and the generation of the large bleb was examined after 10 min. (B) HeLa cells were detached from the substratum by trypsin/EDTA and conditioned medium, and then stimulated by fresh serum. DIC images of the same microscopic field before (left) or after (right) 5 min of treatment with fresh serum are shown. The arrows indicate the large cytoplasmic bleb. (C) Several adherent cell lines were detached from the substratum as in B, stimulated with fresh serum for 3 or 10 min, and fixed. The frequency of the large bleb was scored and summarized as shown. (D and E) HeLa cells were treated with fresh serum for 10 min and fixed. Nuclear lamin B1 was detected by immunofluorescence. White arrows indicate the nuclear bud without lamin B1 that was inside the cytoplasmic large bleb. (F) The frequency of each micronuclei was assessed using the slide shown in D and E. To obtain the data in A, C and F, more than 500 cells were examined in each of three independent scoring processes at each point.

The results described so far show the blebbing and budding phenomena in COLO 320DM cells. These findings were next tested in other cell lines. Because COLO 320DM cells adhere very weakly to the substratum and show a rounded morphology, the experiments were first performed with non-adherent cells. HL-60 and K562 cells weakly formed a large bleb despite treatment with fresh serum, indicating that the non-adherent nature of cells was not linked to the formation of large blebs. Most of the adherent cells tested and listed in Figure 5C did not generate the large bleb in the presence or absence of fresh serum, if they were attached to the substratum (data not shown). However, among these cells, human HeLa cells actively produced the large bleb if the cells were detached from the substratum by trypsin/EDTA, suspended in a conditioned medium, and then stimulated with fresh serum (Figure 5B and C). This requirement for the detachment of cells is consistent with the hypothesis that blebbing is active during mitosis, when adherent cells round up. Concordantly, nuclear budding also became detectable after this procedure (Figure 5D and E). Furthermore, the micronuclei count revealed that the frequency of the lamin-negative micronuclei increased both in the bleb and in total cells (Figure 5F), as in the case of COLO 320DM lines. Human colorectal carcinoma HCT116 or human normal diploid fibroblast WI-38 or WS1 cells also exhibited the blebbing after the detachment from the substratum and the fresh serum stimulation (Figure 5C). However, the nuclear budding was not evident in these cells, and the frequency of the micronuclei did not increase after serum addition (Figure S5). Taken together, the present data suggest that the blebbing and budding are induced by fresh serum only in cells with a specific genetic background.

### CPT induced interphase micronucleation through blebbing and budding

**Figure 6:**
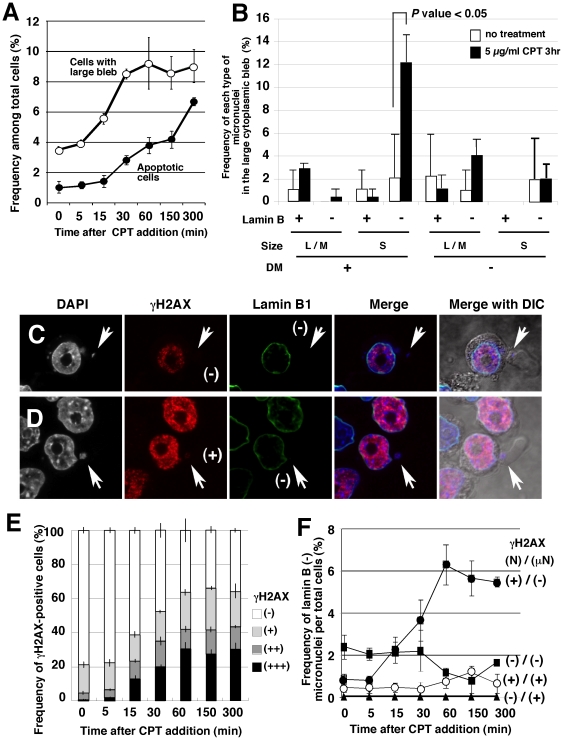
CPT induced interphase micronucleation through blebbing and budding. COLO 320DM-GFP cells were cultured in the presence of 5 µg/ml of CPT for the indicated time (A, E and F) or 3 hours (B to D) and fixed with PFA. Lamin B1 and DM-GFP (B), or lamin B1 and γH2AX (A, C to F) were simultaneously detected by immunofluorescence microscopy. (A) The frequencies of apoptotic cells and cells with large blebs among the total cells were scored and plotted. (B) The frequency of several kinds of micronuclei that were trapped in the large bleb among the cells with large blebs (85 non-treated and 246 CPT-treated) was scored and plotted. (C and D) Representative images of the large bleb trapping the lamin B-negative micronuclei. These micronuclei were either γH2AX-negative (C) or positive (D), while the neighboring nucleus was γH2AX-positive. (E) Time course of the appearance of γH2AX-positive cells. The intensities of the nuclear γH2AX-signal were classified from – to +++ according to a previous report [19]. (F) The lamin B-negative micronuclei were classified according to the distribution of the γH2AX-signal between the nucleus and the micronucleus. To obtain these data, more than 500 (A, E and F) or 1,000 (B) cells were examined in each of three independent scoring processes at each point.

The fresh serum stimulation of the large cytoplasmic bleb described occurred independently from the induction of apoptosis. However, cytoplasmic blebbing is known to be activated during apoptosis. To address this issue, the effect of the apoptotic inducer CPT on blebbing and budding was assessed. Treatment of COLO 320DM-GFP cells with 5 µg/ml CPT induced the formation of large cytoplasmic blebs with a later timing than with fresh serum induction (compare Figure 6A with Figure 5A, C), and faster than the onset of apoptosis (Figure 6A). As a portion of the bleb contained micronuclei, as in the case of serum induction, the frequency of various types of micronuclei in the bleb was examined. CPT predominantly induced the formation of lamin B-negative, small-sized DM-positive micronuclei (Figure 6B). Consequently, the frequency of the lamin B-negative small micronuclei among the total cells greatly increased (Figure S6). On the other hand, as shown in Figure 6B, cytoplasmic blebs with Lamin B-negative, DM-negative and large to medium-sized micronuclei also increased slightly. This finding will be discussed in the following sections.

CPT inhibits topoisomerase I and induces multiple DNA-breaks at replication sites in the S phase nucleus, which can be visualized by the immunofluorescence detection of phosphorylated histone H2AX (γH2AX). Thus, a bright γH2AX signal was detected throughout the nucleus after the addition of CPT (Figure 6C, D). The density of the γH2AX signal in the nucleus varied significantly among the cells, which were therefore classified into 4 categories (– to +++), as described in our previous report [19]. The nuclear γH2AX-positive cells were likely reflect the cells in S phase, and the fraction of γH2AX-positive cells reached more than 50% of total cells during the first 60 min of CPT treatment (Figure 6E). Assessment of the distribution of γH2AX signals between the nucleus and the micronucleus in cells bearing lamin B-negative micronuclei during the CPT treatment time (Figure 6F) showed that CPT induced γH2AX-negative micronuclei that were associated with the γH2AX-positive nucleus. The implications of this finding will be discussed.

### Blebbing and budding were most frequent during S phase

**Figure 7:**
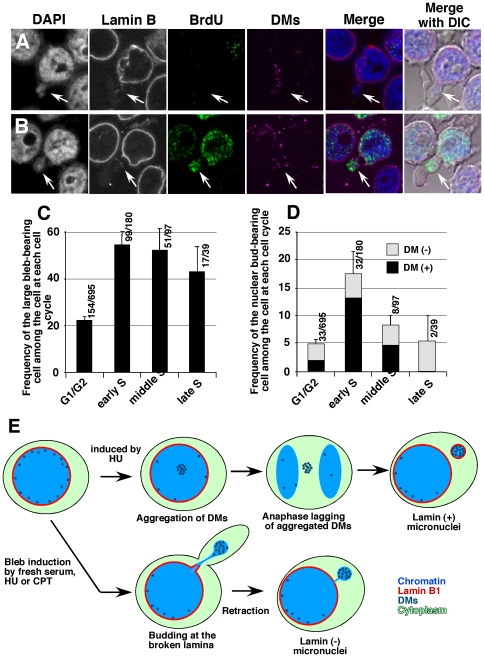
Cell cycle dependency and graphical summary of the blebbing and budding. (A and B) BrdU (20 µg/ml) was added to the logarithmically growing culture of COLO 320DM-GFP cells. After 5 min, fresh serum was added at 10% concentration, and the cells were harvested 10 min later and fixed with PFA. The lamin B protein and BrdU were detected by immunofluorescence, and DMs were detected by FISH. Two representative cells showing blebbing and budding are shown. The cell cycle phase was determined by the nuclear distribution of BrdU [24]; the cell in A is non-S and the cell in B is at the early S phase. (C) The frequency of the large bleb-bearing cells among the cells at each cell cycle phase was scored, which was obtained by examining the number of cells shown in the denominator noted at each bar. (D) The frequency of the DM-positive or negative nuclear bud-bearing cells among the cells at each cell cycle phase is shown. (E) Model showing the generation of the different types of micronuclei as revealed by the findings of the study.

Based on previous findings showing that nuclear budding is frequent during the S phase [24], the effect of cell cycle stage on blebbing/budding was assessed. Logarithmically growing COLO 320DM-GFP cells were labeled with BrdU for 15 min and stimulated with serum for 10 min. The cells were fixed and the incorporated BrdU was detected by immunofluorescence microscopy, which allowed the determination of the cell cycle phase of each cell according to the nuclear distribution of the incorporated BrdU [24]. Figure 7A and B show representative images of the lamin B-negative bud in the cytoplasmic bleb that was formed in non-S or early S phase cells, respectively. The frequency of the cytoplasmic blebs (Figure 7C) was low among the non-S phase cells, and it was two-fold higher in the S phase cells, with the highest frequency in early S phase cells. Concordantly, the frequency of the nuclear buds (Figure 7D) was low among the G1/G2 cells, and it was highest in the early S phase cells. In particular, buds bearing DMs were remarkably induced in the early S phase cells.

## Discussion

### Micronucleation through blebbing and budding

The present study describes the unanticipated finding that nuclear budding/micronucleation is coupled with cytoplasmic membrane blebbing. This coupling was directly shown by the careful examination of fixed cells and the time-lapse examination of live cells. Furthermore, our findings suggest that cytoplasmic blebbing may cause nuclear budding, as suggested by the induction of nuclear budding in response to the stimulation of cytoplasmic blebbing by two unrelated stimuli, fresh serum and CPT. In addition, conditions that induced blebbing and budding also induced the formation of lamin-negative micronuclei, indicating that micronuclei are generated by nuclear budding. Taken together, the present results suggest a surprising mechanism by which the cytoplasmic membrane dynamics may pull the chromatin out of the nucleus.

The described mechanism generated predominantly lamin-negative micronuclei. Because transcription [22] and replication (A. Okamoto et al., unpublished data) do not proceed in such micronuclei, incorporation of genetic material into these structures has important implications for the genetic diversification of cancer cells. Interestingly, it was reported that the nuclear bleb-shaped structures became frequent after inhibiting the expression of either lamin A [30], [31] or B1 protein [32]. Such nuclear bleb-shaped structures were devoid from either lamin B1 or B2, respectively.

### Coupling of nuclear budding to cytoplasmic blebbing

Prior studies suggested that local osmolarity changes might generate hydrostatic pressure inside the cell, which in turn could cause the formation of cytoplasmic protrusions [33], [34]. According to this hypothesis, hydrostatic pressure could pull the chromatin out of the nucleus if the nuclear lamina is partly broken, as illustrated in Figure 7E. In our study, the nuclear bud was not induced in normal cells by the serum, despite the formation of large cytoplasmic blebs (Figure 5 and S5), suggesting that the lamina structure was intact in these cells. The blebbing and budding may occur only in a population of tumor cells with an abnormal lamina structure.

The present finding that the blebbing and budding occurs frequently during the S phase is consistent with findings reported previously [24]. This phenomenon might be explained by the increase in the nuclear volume during the S phase, as the amount of genetic material doubles. The lamin B protein is synthesized after the initiation of S phase, while lamin A or C is synthesized throughout the cell cycle [35]. If the synthesis of lamin B protein cannot compensate for the increase in the nuclear volume, the lamina may be partly broken, which would explain the increased frequency of nuclear budding during S phase.

### Induction of blebbing and budding

The induction of cytoplasmic blebbing by fresh serum has been reported previously [28], [29]. Fresh serum acts as a growth promoting signal, and many serum-response factors have been found. Serum response factors were suggested as master regulators of the actin cytoskeleton and contractile apparatus [36]. Importantly, the actin cytoskeleton is frequently linked to the regulation of cytoplasmic blebbing [37]–[40], suggesting a possible connection between serum incubation and the induction of blebbing and budding.

On the other hand, CPT was shown to also induce blebbing and budding (Figure 6). CPT is a topoisomerase I inhibitor that causes DNA double strand breakage in S phase nuclei, and the damage signal may elicit the blebbing that is observed during the early stages of apoptosis. This is consistent with the finding that hydroxyurea (HU) treatment also induced both DNA damage [19] and interphase micronucleation (Figure S7), albeit at a low frequency. Our findings are also consistent with a report describing the elimination of Rad 51-positive chromatin from the nucleus during interphase after treatment with gamma-irradiation [41]. Interestingly, CPT induced the formation of γH2AX-negative buds in the cell, while the nucleus was γH2AX-positive (Figure 6F). This finding could imply that the initial local DNA damage may elicit a signal leading to cytoplasmic blebbing, and then the entire nucleus might become γH2AX-positive. The buds thus formed might remain γH2AX-negative due to the absence of the lamina that is required for normal nuclear function. This hypothesis needs to be addressed in future work.

### Mechanisms that eliminate the extrachromosomal element

Our previous work [19] together with the current work showed that micronuclei enriched with DMs were generated after mitosis from DM aggregates, which are lamin B1-positive. The results of the present study showed that blebbing and budding also generate lamin-negative micronuclei. These lamin-negative micronuclei contained DMs more frequently than the chromosomal materials, which could be explained by that DMs are acentric extrachromosomal chromatin, which is smaller than the normal chromosome. Furthermore, DMs frequently localize to the nuclear periphery until early S phase [17]. Therefore, it is reasonable to assume that the small DMs at the nuclear periphery may easily pass through the small breaks in the nuclear lamina. On the other hand, the present findings suggested that the extrusion of the chromosome material required large lamina breaks. This is consistent with prior findings showing that interstitial DNA without a centromere or telomere is more prevalent in nuclear buds that are connected to the nucleus than in micronuclei that are apart from the nucleus [42]. The latter should be formed after mitosis, while the former might be formed by the interphase budding.

In a previous report, microinjected DNA was shown to be rapidly aggregated in the nucleus. Surprisingly, part of this aggregate was found to pass through the nuclear rim and move to the cytoplasm of the living cell [43]. The stress caused by the microinjection might induce blebbing, which could draw the aggregate out of the nucleus through the small lamina break.

### Conclusion

The present study suggests a novel mechanism by which cytoplasmic membrane dynamics may pull the chromatin out of the nucleus. This phenomenon depends on the intensity of cytoplasmic blebbing and the integrity of the nuclear lamina, and the process may eliminate the extrachromosomal genetic material that plays a crucial role in the establishment of the malignant phenotype. This mechanism could therefore have a profound influence in the determination of the cancer cell phenotype.

## Materials and Methods

### Cell lines and culture

Human colorectal tumor COLO 320DM (CCL 220) cells [11], chinese hamster CHO-K1 cells [44], human leukemia HL-60 cells [45], K562 cells [45], and human cervical tumor HeLa cells [3] were obtained and maintained as described previously. The human colorectal carcinoma HCT116 cell line was a kind gift from Dr. B. Vogelstein (Johns Hopkins University).

The COLO 320DM-GFP cells were previously established from COLO 320DM cells [19]. Namely, the DMs were tagged by amplifying lactose operator (LacO) arrays on DMs using a novel method, and visualized by the expression of the lactose repressor (LacR)-GFP protein. The COLO 320DM-GFP/lamin B1-mCherry cells were established as follows. The pLamin B1-GFP (Neo) plasmid [46], which was a kind gift from Robert D. Goldman, was digested with *Nhe* I and *Bsp* E1 to excise the EGFP gene. The plasmid pAWS-mCherry was obtained from EUROSCARF, and the mCherry gene was amplified using a primer bearing a 15 bp sequence that flanks the *Nhe* I and *Bsp* E1 digested ends of the pLamin B1-GFP plasmid. The plasmid pLamin B1-mCherry (Neo), in which the original EGFP gene was substituted by the mCherry gene, was produced using the In-Fusion advantage PCR cloning kit (Clontech Co). The neomycin-resistant gene (neo) was replaced by the blasticidine resistant gene (BS) and the ampicillin resistant gene (Amp). For this substitution, the pLamin B1-mCherry (Neo) was digested by *Nae* I and the BS/Amp expression cassettes were PCR-amplified from the pSFVdhfr plasmid [47]. The latter was cloned in the former vector by using the In-Fusion reaction, and the pLamin B1-mCherry (BS) was obtained. This plasmid was transfected into Neomycin-resistant COLO 320DM-GFP cells by lipofection, and selected by blasticidine. A cell clone showing both the bright lamin B1-mCherry signal at the nuclear rim and the many bright GFP-labeled DMs was isolated and used in this study.

Hydroxyurea (HU; Sigma) or camptothecin (CPT; Sigma) were added to the culture at 100 µM or 5 µg/ml, respectively.

### Serum stimulation, cell fixation and the cytochemical procedure

Logarithmically growing COLO 320DM cells, COLO 320DM-GFP cells, COLO 320DM-GFP/lamin B1-mCherry cells or other non-adherent cells (HL-60 and K562) in 3 cm dish were stimulated by adding 1/10 volume of fresh serum (Euroclone co., Italy). The cells were cultured for an additional 10 min, and directly fixed by adding an equal volume of 4% PFA followed by incubation at room temperature for 10 min. Adherent cells (HeLa, CHO-K1 and HCT116) were detached from the substratum and suspended in the conditioned medium. The conditioned medium was prepared by centrifuging a 3 day old culture of the same cell line. The detached adherent cells were stimulated by fresh serum and fixed as described above. The fixed cells were used for counting the frequency of the cytoplasmic blebbing. For the immunological detection of the lamin B1 protein, the PFA-fixed cells were collected onto a slide glass by cyto-centrifugation. Because the cells with large cytoplasmic blebbing were easy to detach from the slide glass, the cyto-centrifuged cells were re-fixed with 4% PFA in PBS for 10 min at room temperature. The cells on the slide were treated with 0.5% NP-40 in PBS- for 10 min, washed with PBS-, immersed in 70% ethanol/water for 5 min, and immersed in PBS-. Lamin B1 was detected by using a goat anti-lamin B1 (C-20) antibody (Santa Cruz Biotechnology, Inc.) and a Texas red–conjugated rabbit anti-goat IgG antibody (EY Laboratories, Inc.), as described previously [22], or FITC-conjugated donkey anti-goat antibody (Santa Cruz Biotechnology Inc.). Phosphorylated histone H2AX (γH2AX) was detected using a mouse IgG antibody (Upstate Biotechnology Inc.) and Alexa 568-conjugated goat anti-mouse IgG (Molecular Probe Inc.). BrdU (Sigma Co.) was detected with a rat monoclonal anti BrdU antibody (oxford biotechnology) and Alexa 488-conjugated goat anti rat IgG (Molecular probe). Apoptotic cells were detected by staining the nucleus in the PFA-fixed cells with DAPI. We counted the cells with fragmented and condensed nuclei as apoptotic cells. For the experiment shown in Figure 7A to D, DMs were detected by FISH using a DIG-labeled probe that hybridized to the plasmid sequence on DMs, and it was detected by a mouse monoclonal anti DIG antibody (Roche) and Alexa 647-conjugated anti mouse IgG (Molecular Probes., Inc.) The FISH was performed by our published protocol [16], and it was applied to the nuclei that were fixed by 3% parafolmaldehyde (PFA) for 10 min at room temperature.

### Microscopy and time-lapse observation of live cells

Time-lapse observations appearing in Figure 1E and 4 were done using the method described previously [3] with some modifications. In brief, the cells were cultured in collagen coated glass bottom dishes (35 mm diameter, MatTek Co., Ashland, MA). For the detection of chromatin in live cells, Hoechst33342 (Calbiochem, Co.) was added to the culture at 100 µg/ml. The dish was then incubated for 30 min, and washed 2 times with the conditioned medium. The cells in the dish were cultured on the microscope stage by using a stage top incubator system (ONIVF; Tokai Hit Co., Japan) with a control unit (INU; Tokai Hit Co.) in a constant flow of air containing 5% CO_2_ at 37°C with constant humidity. The incubator system was equipped with a Nikon inverted microscope (TE2000-E, Nikon, Tokyo). Both the microscope and the CCD camera (DS, Nikon) were controlled by NIS-element software (Nikon). The differential interference contrast (DIC) images and the epifluorescence images were captured using the precentered Fiber Illuminator (Intensilight, C-HGFI, Nikon), the ND-filter, the adequate fluorescence filter set, and objective lens (Nikon Plan Apo VC 60×/1.40 Oil). In most experiments, six to eight images at 0.8∼1.4 µm z-intervals were captured at 5 to 30 min time intervals. The acquired images were viewed and deconvolved using the NIS-element software. Other images were obtained by using an Olympus FV10-ASW confocal system on FV1000D-IX81 with an x60 objective (UPLSAPO NA 1.35_60 oil). All images were processed using Adobe Photoshop CS version 8.0.1 (Adobe Systems Inc.).

## Supporting Information

Figure S1 **Fresh serum stimulation of cytoplasmic blebbing did not increase the apoptotic cell frequency.**
(TIF)

Figure S2 **Live cell time-lapse examination of the blebbing and budding.** COLO 320DM-GFP cells were examined. For bright filed images, phase contrast images were obtained. The arrows indicate the blebbing and budding.
(TIF)

Figure S3 **Live cell time-lapse examination of the blebbing and budding.** COLO 320DM-GFP cells were examined. For bright filed images, DIC images were obtained. The arrows indicate the blebbing and budding. The complete movie appears in the Movie S1.
(TIF)

Figure S4 **Live cell time-lapse examination of the blebbing and budding.** COLO 320DM-GFP/Lamin B1-mCherry cells were examined. For bright filed imaging, DIC images were obtained. The arrow indicates the blebbing and budding.
(TIF)

Figure S5 **Frequency of each micronuclei after fresh serum stimulation in several cell lines.**
(TIF)

Figure S6 **CPT induced an increase in lamin B-negative small-sized micronuclei in COLO 320DM-GFP cells.**
(TIF)

Figure S7 **HU as well as CPT induced interphase micronucleation.** The image shows the summary of many time-lapse experiments in the presence of 100 µM HU or 5 µg/ml CPT.
(TIF)

Movie S1 **Live cell time-lapse examination of the blebbing and budding.** COLO 320DM-GFP cells were examined. For bright filed images, DIC images were obtained. The arrows indicate the blebbing and budding.
(MOV)
