# Supplementary figures and images for: Generation of Micronuclei during Interphase by Coupling between Cytoplasmic Membrane Blebbing and Nuclear Budding
Source: PLoS One. 2011 Nov 2;6(11):e27233. doi: 10.1371/journal.pone.0027233 (PMC3206950; doi:10.1371/journal.pone.0027233)

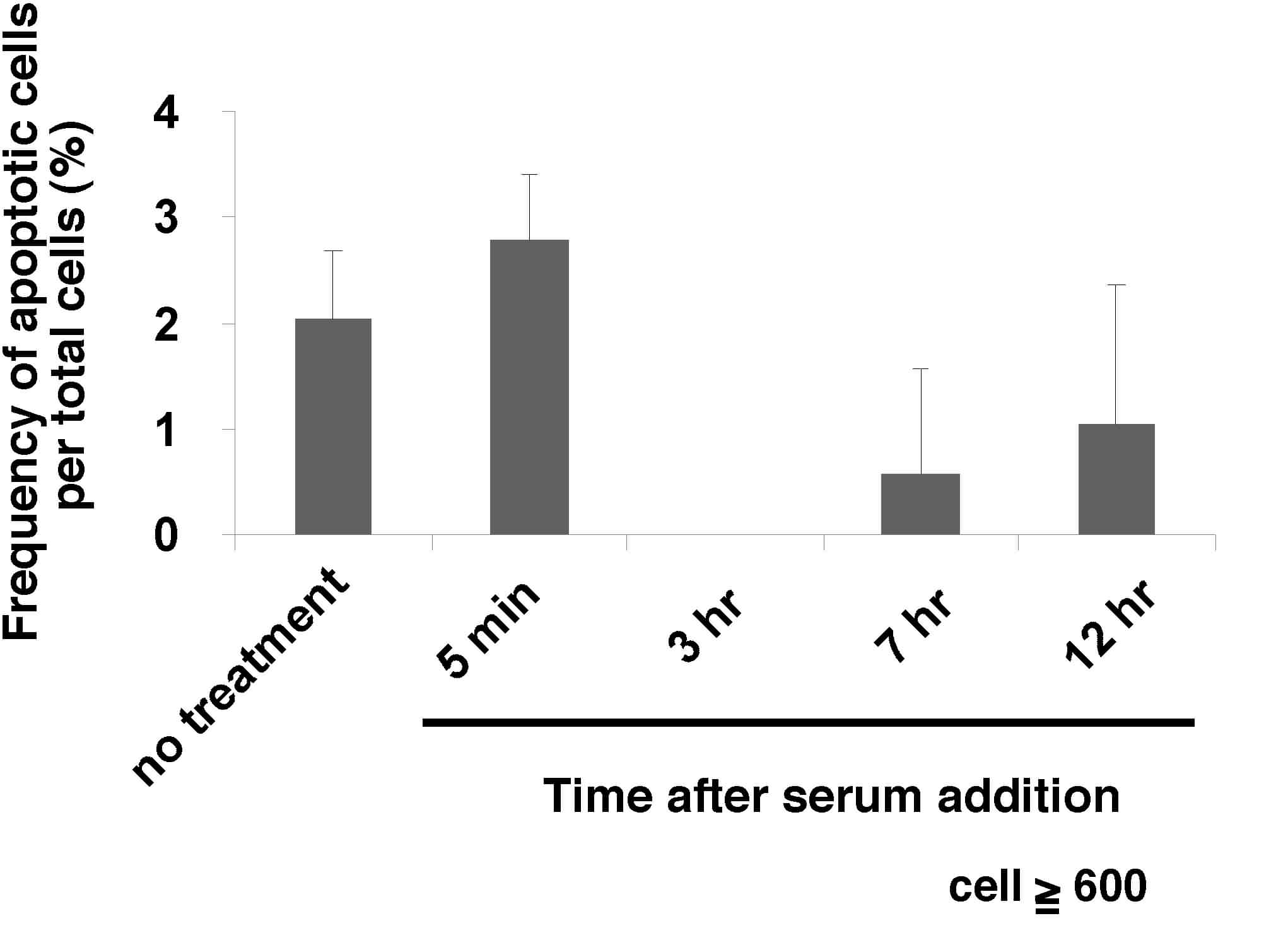

Supplement: Figure S1 — Fresh serum stimulation of cytoplasmic blebbing did not increase the apoptotic cell frequency. (TIF) [file pone.0027233.s001.tif]

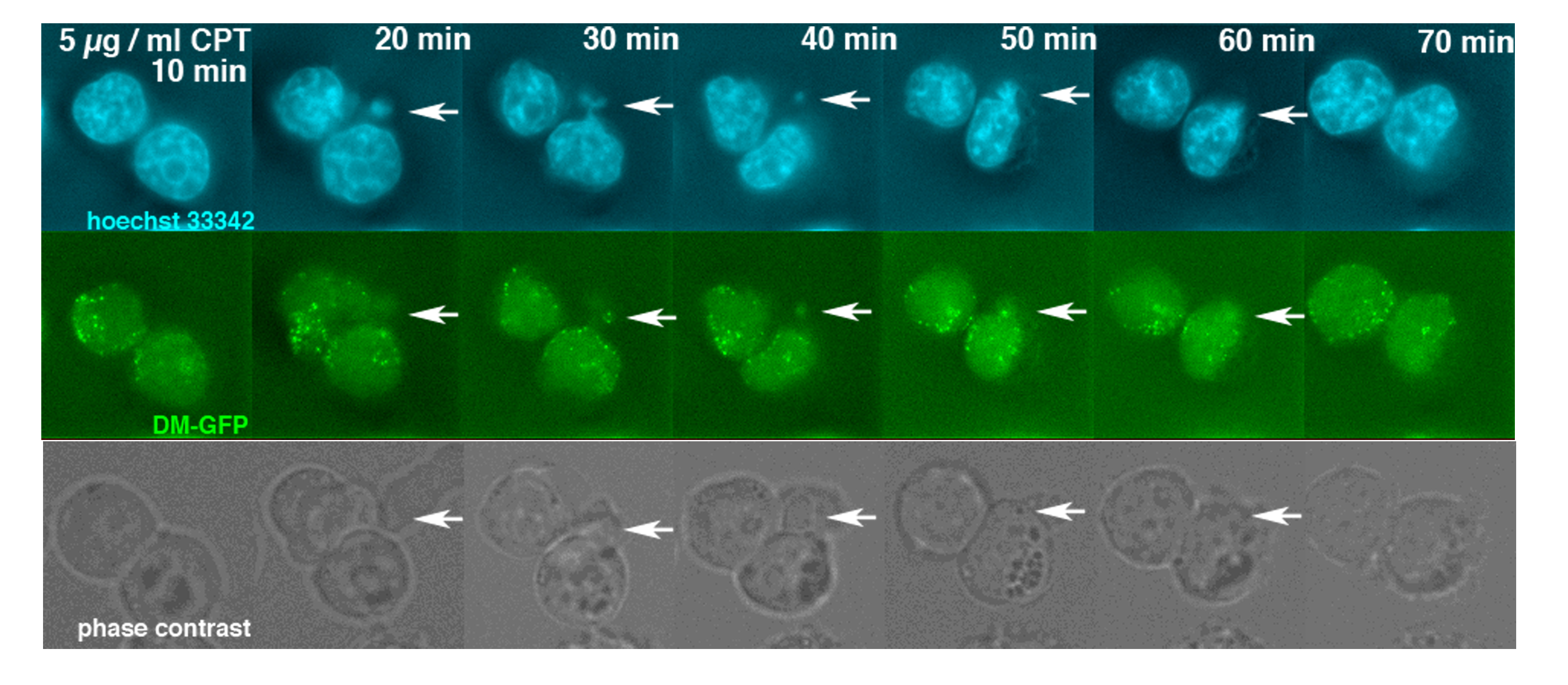

Supplement: Figure S2 — Live cell time-lapse examination of the blebbing and budding. COLO 320DM-GFP cells were examined. For bright filed images, phase contrast images were obtained. The arrows indicate the blebbing and budding. (TIF) [file pone.0027233.s002.tif]

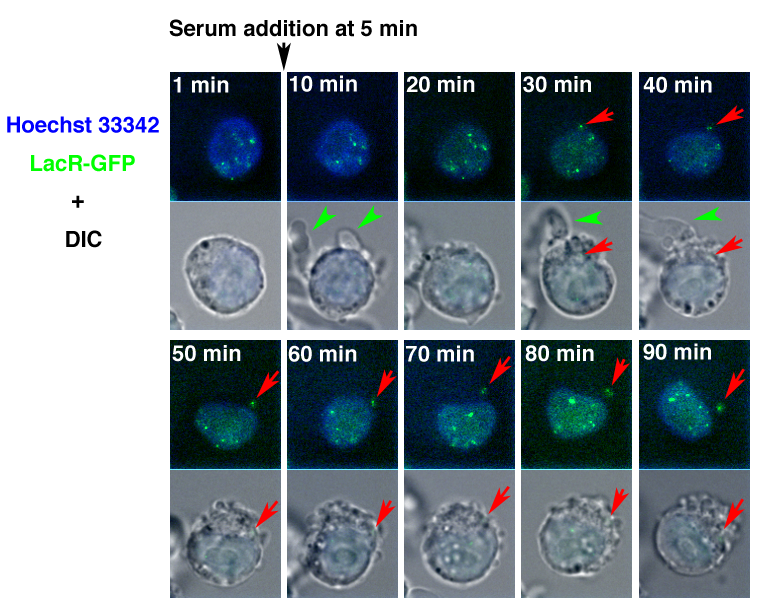

Supplement: Figure S3 — Live cell time-lapse examination of the blebbing and budding. COLO 320DM-GFP cells were examined. For bright filed images, DIC images were obtained. The arrows indicate the blebbing and budding. The complete movie appears in the Movie S1. (TIF) [file pone.0027233.s003.tif]

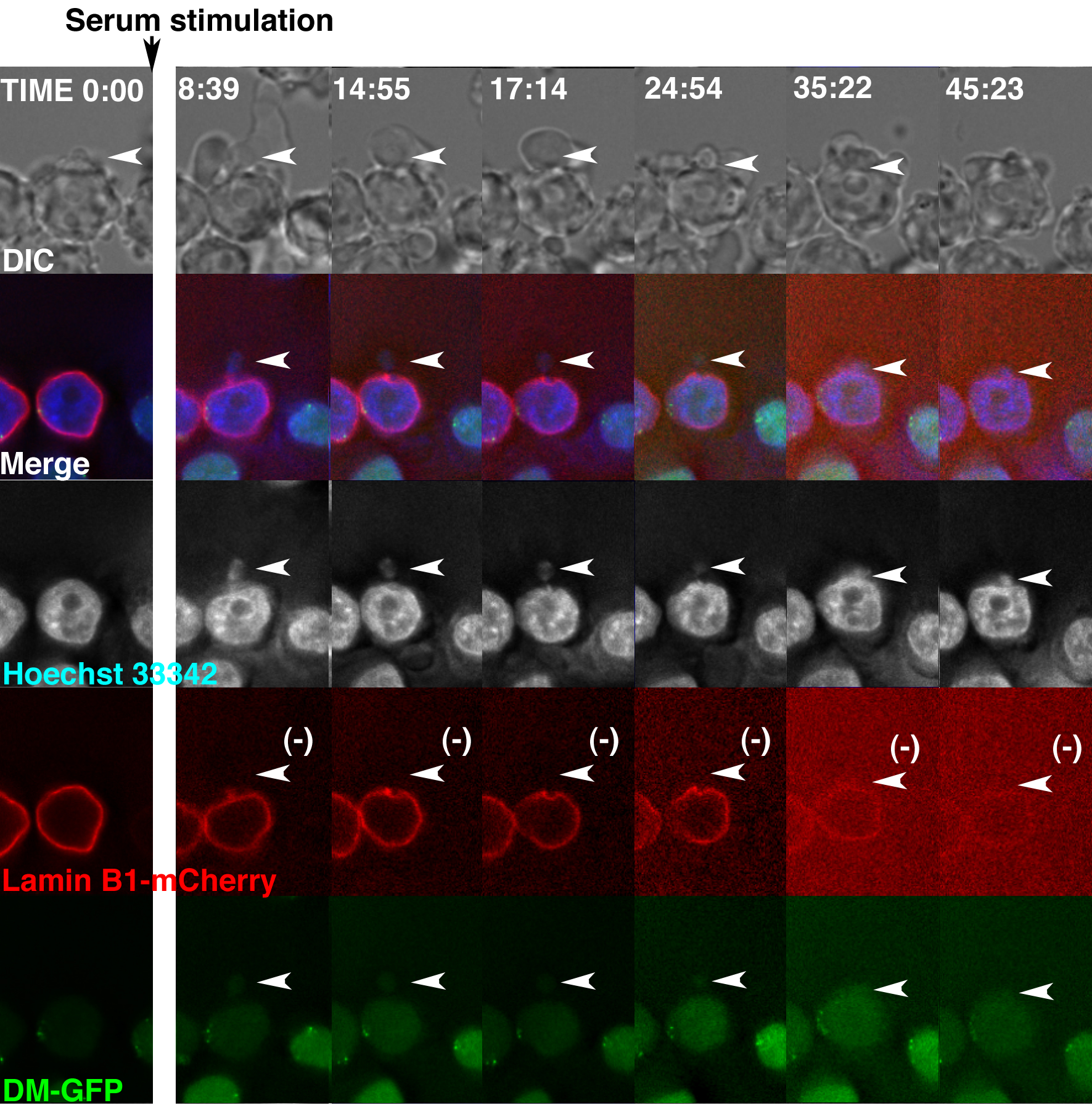

Supplement: Figure S4 — Live cell time-lapse examination of the blebbing and budding. COLO 320DM-GFP/Lamin B1-mCherry cells were examined. For bright filed imaging, DIC images were obtained. The arrow indicates the blebbing and budding. (TIF) [file pone.0027233.s004.tif]

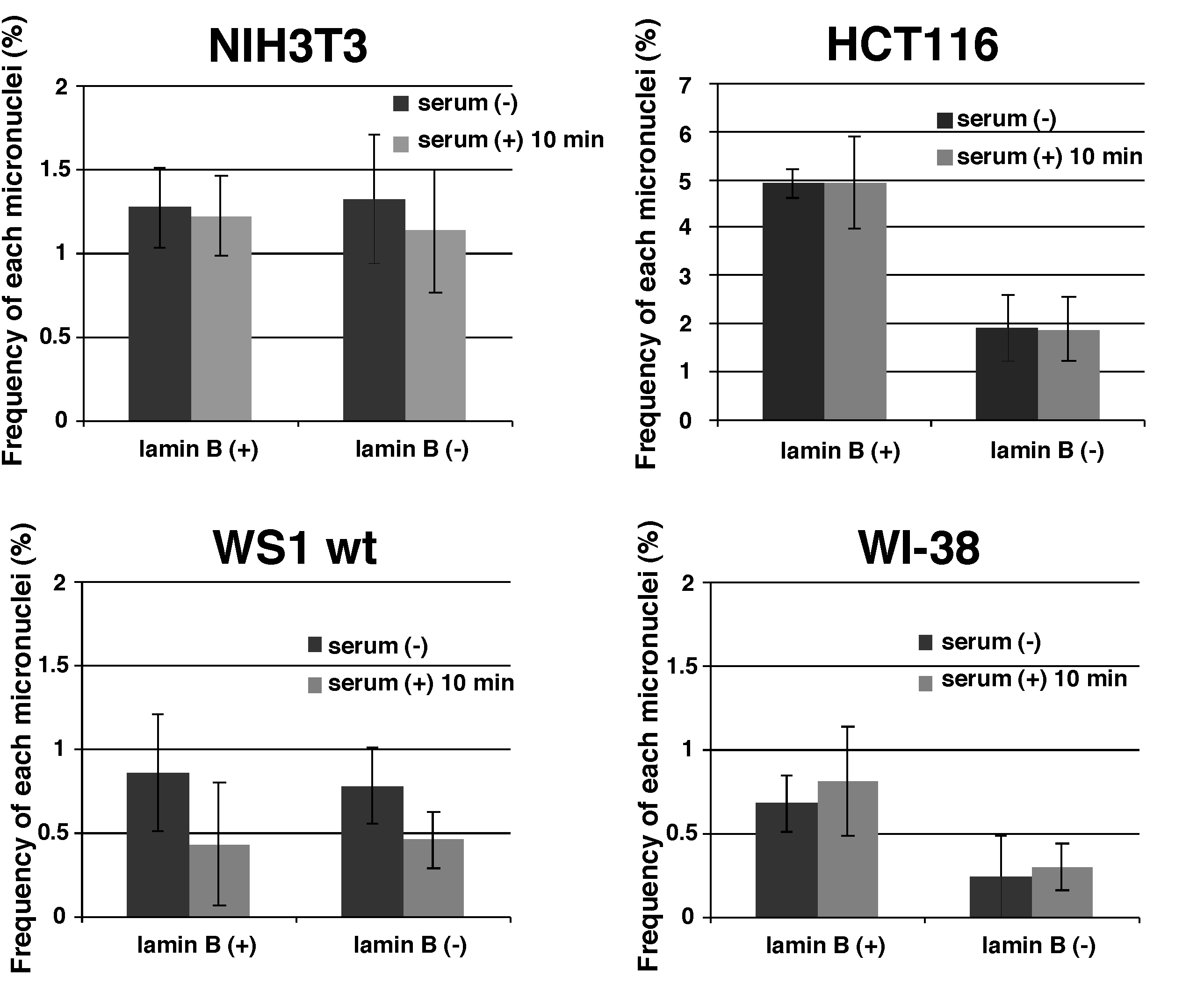

Supplement: Figure S5 — Frequency of each micronuclei after fresh serum stimulation in several cell lines. (TIF) [file pone.0027233.s005.tif]

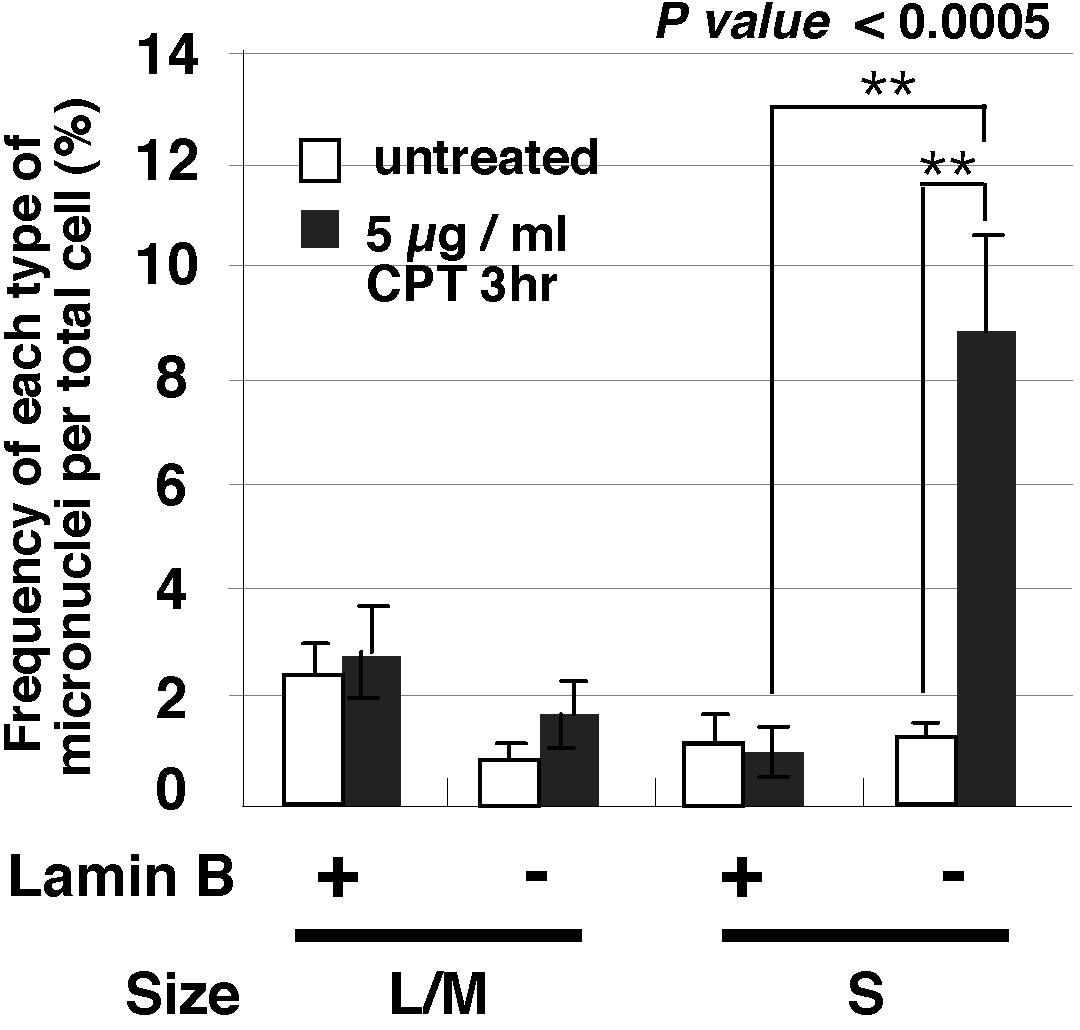

Supplement: Figure S6 — CPT induced an increase in lamin B-negative small-sized micronuclei in COLO 320DM-GFP cells. (TIF) [file pone.0027233.s006.tif]

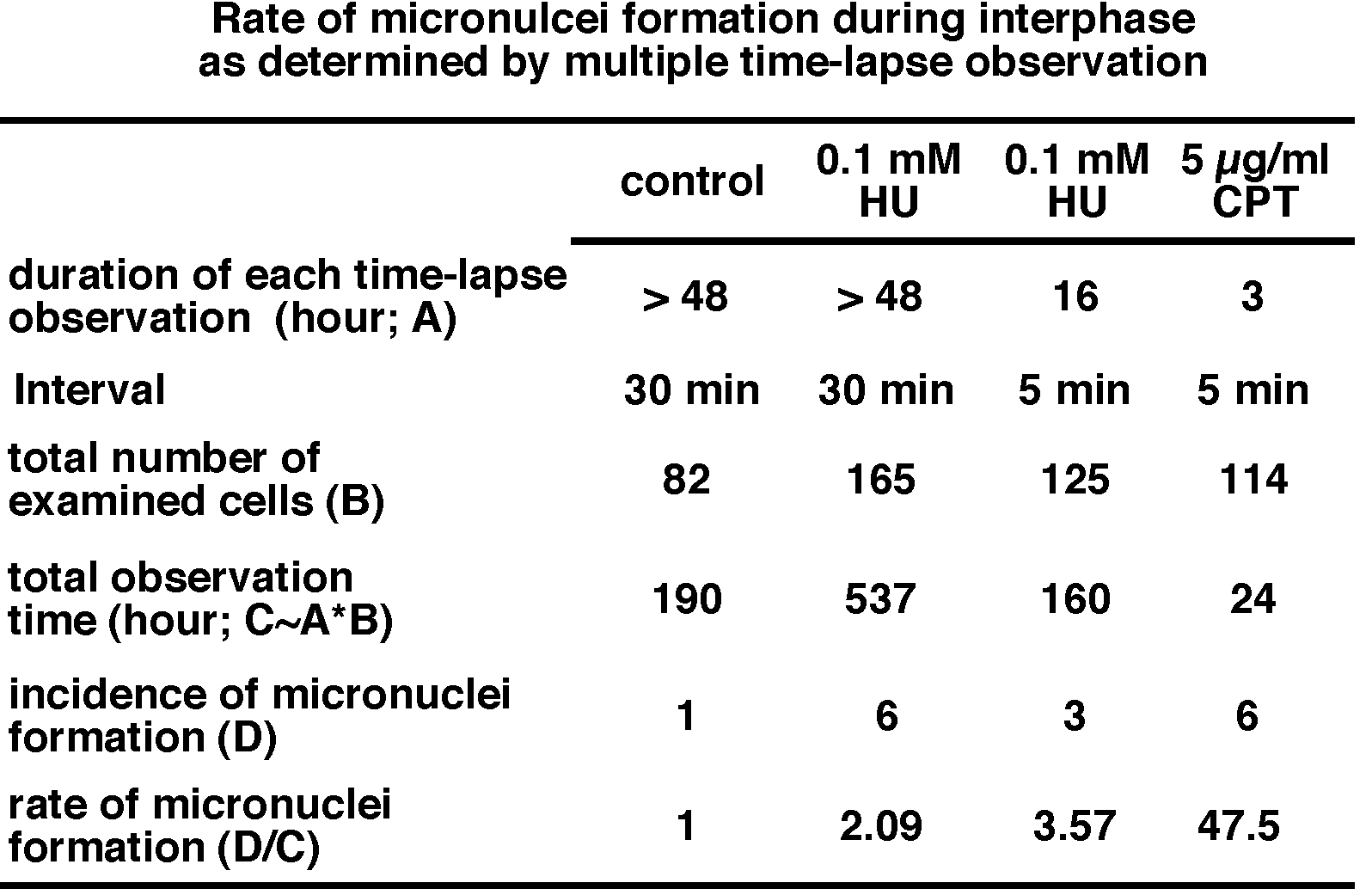

Supplement: Figure S7 — HU as well as CPT induced interphase micronucleation. The image shows the summary of many time-lapse experiments in the presence of 100 µM HU or 5 µg/ml CPT. (TIF) [file pone.0027233.s007.tif]
